# Immune Checkpoint Blockade in Lung Carcinoids with Aggressive Behaviour: One More Arrow in Our Quiver?

**DOI:** 10.3390/jcm11041019

**Published:** 2022-02-16

**Authors:** Sergio Di Molfetta, Tiziana Feola, Giuseppe Fanciulli, Tullio Florio, Annamaria Colao, Antongiulio Faggiano, NIKE Group

**Affiliations:** 1 Section of Internal Medicine, Endocrinology, Andrology and Metabolic Diseases, Department of Emergency and Organ Transplantation, University of Bari Aldo Moro, 70124 Bari, Italy; s.dimolfetta@libero.it; 2 Department of Experimental Medicine, “Sapienza” University of Rome, 00161 Rome, Italy; tiziana.feola@uniroma1.it; 3 Neuroendocrinology, Neuromed Institute, Scientific Institute for Research, Hospitalization and Healthcare, 86077 Pozzilli, Italy; 4 NET Unit, Department of Medical, Surgical and Experimental Sciences, University of Sassari—Endocrine Unit, University Hospital of Sassari, 07100 Sassari, Italy; 5 Department of Internal Medicine, University of Genoa, 16132 Genoa, Italy; tullio.florio@unige.it; 6 Scientific Institute for Research, Hospitalization and Healthcare Ospedale Policlinico San Martino, 16132 Genoa, Italy; 7 Endocrinology Unit, Department of Clinical Medicine and Surgery, University Federico II, 80131 Naples, Italy; colao@unina.it; 8 Endocrinology Unit, Department of Clinical and Molecular Medicine, Sant’Andrea Hospital, Sapienza University of Rome, 00189 Rome, Italy; antongiulio.faggiano@uniroma1.it

**Keywords:** typical lung carcinoid, atypical lung carcinoid, neuroendocrine neoplasms of the lung, immune checkpoint inhibitors, immunotherapy

## Abstract

Lung carcinoids are well-differentiated and low-/intermediate-grade neuroendocrine neoplasms of the lung. Given their relative rarity, and the paucity of data available from prospective studies, no global consensus exists on the systemic treatment of these tumours. In recent years, immune checkpoint inhibitors have revolutionized cancer management and are under evaluation in patients with diverse types of neuroendocrine neoplasms. The aim of this narrative review is to analyse all available data for the use of approved immune checkpoint inhibitors in patients with lung carcinoids. We performed an extensive search for relevant data sources and found five published articles, one meeting abstract, and nine registered clinical trials indicating a growing interest of researchers in this field, and providing preliminary evidence of efficacy for combined nivolumab plus ipilimumab and durvalumab plus tremelimumab regimens in the treatment of advanced and/or metastatic lung carcinoids.

## 1. Introduction

Neuroendocrine neoplasms (NENs) of the lung comprise a heterogeneous group of tumours that are subdivided, according to the World Health Organization (WHO) classification, into four subtypes: typical carcinoid (TC); atypical carcinoid (AC), collectively named lung carcinoids (LCs); small cell lung cancer (SCLC); and large cell neuroendocrine carcinoma [1]. LCs are well-differentiated (WD) and low-/intermediate-grade lung NENs [1], with TC accounting for 90% of all LCs [2].

While most LCs show a favourable outcome, their clinical course may be quite heterogeneous, ranging from no/minimal progression to aggressive (rapidly progressive/metastatic) behaviour [3].

Surgery is the reference standard of treatment for LCs with loco-regional disease [4]. For advanced or unresectable tumours, several therapeutic options are available, including somatostatin analogues [5], peptide receptor radionuclide therapy (PRRT) [6], chemotherapy (doxorubicin, dacarbazine, 5-fluorouracil, capecitabine, cyclophosphamide, streptozotocin, platinum derivatives, etoposide, and temozolomide) [7], mTOR inhibitors (everolimus) [8], and tyrosine kinase inhibitors (pazopanib) [9].

Guidelines for the management of advanced LCs have been proposed by international scientific societies such as the European Neuroendocrine Tumour Society (ENETS), the National Comprehensive Cancer Network (NCCN), and the Commonwealth Neuroendocrine Tumour Research Collaboration (CommNETs) with the North American Neuroendocrine Tumour Society (NANETS) [4,6,10]. However, given the relative rarity of these tumours, and the few data available from prospective studies, no global consensus exists on the systemic treatment of LCs.

In recent years, monoclonal antibodies targeting programmed cell death protein 1 (PD-1), programmed death ligand 1 (PD-L1), and cytotoxic T lymphocyte associated protein 4 (CTLA-4), hereinafter collectively referred to as immune checkpoints inhibitors (ICIs), have been added to the therapeutic arsenal and have revolutionized cancer management. Indeed, tumour cells are able to escape immune cell recognition and the subsequent immune-mediated cytotoxicity, through the impairment of different systems that normally make them vulnerable to effector T cells [11]. These include (i) the downregulation of MHC class I antigens, (ii) the triggering of negative feedback mechanisms mediated by inhibitory cytokines (IL-10 and TGF-β), (iii) the activation of immunosuppressive cell populations (regulatory T and B cells, Tregs, and Bregs), and, above all, (iv) the stimulation of immune inhibitory receptors such as CTLA-4 and PD-1.

CTLA-4 is a negative regulator of T cells primarily acting within lymphoid tissues, while the PD-1/PD-L1 system is mainly active in tissues where the immune response is on-going, including tumours [12]. Thus, through the blockade of CLTLA-4 and/or the PD-1/PD-L1 system, ICIs have provided a novel mechanism for treating cancer, that is reactivation of the immune-mediated tumour killing [13,14], which is associated, from a clinical point of view, with durable responses and a favourable safety profile in patients for whom other cancer therapies have failed [15]. A comprehensive list of all agents being approved for human use and their indications is provided in Table 1.

Given the broad spectrum of the efficacy and the favourable safety profile, ICIs are currently under evaluation in patients with NENs [16,17,18,19,20,21,22]. Of note, a minority of LCs express PD-L1 [23,24], and PD-L1 expression is sometimes associated with metastatic potential [23] and poorer survival [24,25]. Vesterinen et al. also found a high PD-1 expression (i.e., >2 PD-1 positive intratumoral lymphocytes per mm^2^) in 16% of LCs [23].

### Aim of the Study

The aim of this narrative review is to analyse all available data on the use of approved ICIs in patients with LCs.

## 2. Materials and Methods

We performed an extensive search for relevant data sources, including (i) full published articles in international online databases (PubMed, Web of Science, Scopus, and Embase), preliminary reports in selected international meeting abstract repositories (American Society of Clinical Oncology (ASCO), European Neuroendocrine Tumour Society (ENET), European Society for Medical Oncology (ESMO)), or short articles published as supplements of scientific journals, and (ii) registered clinical trials (RCTs) in the U.S. National Institutes of Health registry of clinical trials (http://clinicaltrials.gov) and in any primary register of the WHO International Clinical Trials Registry Platform (ICTRP). The following keywords were used: typical lung carcinoid, atypical lung carcinoid, immune checkpoint inhibitors, immunotherapy, CTLA-4, PD-L1, PD-1, atezolizumab, avelumab, camrelizumab, cemiplimab, durvalumab, ipilimumab, nivolumab, pembrolizumab, penpulimab, sintilimab, tislelizumab, toripalimab, and zimberelimab. The search was last updated on 19 December 2021.

## 3. Results

### 3.1. Published Articles

We found 63 published articles, of which five were relevant to the study aim. Reasons for article exclusion were as follows: (i) duplicated article, (ii) article not written in English, (iii) study on animal models, (iv) data on non-lung cancers, (v) general review on lung cancers, (vi) data shown as cumulative response with other NENs, and (vii) article not dealing with clinical issues. We also found one preliminary report in international meeting abstract repositories.

### 3.2. Registered Clinical Trials (RCTs)

We found 108 RCTs, nine of which were ongoing and matched the aim of the study (Table 2). We only identified phase II trials, one with atezolizumab and bevacizumab (active, not recruiting), one with durvalumab and tremelimumab (recruiting), two with ipilimumab in combination with nivolumab (active, not recruiting), one with nivolumab and cabozantinib (recruiting), one with nivolumab with stereotactic body radiation therapy (recruiting), one with nivolumab and temozolomide (active, not recruiting), one with pembrolizumab (recruiting), and one with tislelizumab in combination with surufatinib (recruiting).

### 3.3. Immune Checkpoint Inhibitors in Monotherapy

The multi cohort, phase 1 KEYNOTE-028 study (NCT02054806) evaluated the activity and safety of the anti–PD1 pembrolizumab at a dose of 10 mg/kg IV every 2 weeks in patients with PD-L1-positive, locally advanced/metastatic carcinoid (irrespective of the site of origin) or well-moderately differentiated pancreatic neuroendocrine tumours (NETs) for up to 2 years. Computed tomography (CT) or magnetic resonance imaging was performed every 8 weeks for the first 6 months of treatment, and every 12 weeks thereafter to evaluate the response as per the Response Evaluation Criteria in Solid Tumours (RECIST) 1.1 criteria. Overall, nine patients with LCs were enrolled in the trial. Although no comprehensive information is provided for this subcohort of patients, none of them showed an objective response upon imaging [26].

NCT02628067 (A Clinical Trial of Pembrolizumab (MK-3475) Evaluating Predictive Biomarkers in Subjects with Advanced Solid Tumours (KEYNOTE 158)) is a study in which patients with multiple types of advanced (unresectable and/or metastatic) solid tumours, including LCs, are treated with pembrolizumab after progression on standard of care therapy. The study includes two cohorts. The first receives pembrolizumab 200 mg intravenously (IV) on day 1 of each 3-week cycle for up to 35 administrations (up to approximately 2 years of treatment). The second consists of any advanced solid tumour that has failed at least one line of therapy and tumour mutational burden (TMB)-high, excluding participants with mismatch repair deficient tumours. The dosing regimen for the latter cohort is 400 mg IV every 6 weeks for up to 18 administrations (up to approximately 2 years of treatment). The primary outcome is the efficacy evaluated through the objective response rate, defined as the percentage of participants who have a complete response (CR) per RECIST 1.1, modified to follow a maximum of 10 target lesions and a maximum of five target lesions per organ at any time during the trial. The duration of response (DOR), progression free survival (PFS), and overall survival (OS) are assessed as secondary measures. The study started in December 2015, with an estimated enrolment of 1100 subjects. The actual status is “recruiting”, with an estimated completion date of June 2026.

### 3.4. Dual Immune Checkpoint Inhibition

The DUNE trial (NCT03095274) is a multi-cohort phase II study evaluating the combination durvalumab plus tremelimumab for the treatment of patients with advanced NENs of either a gastroenteropancreatic (GEP) or lung origin. The preliminary results of this trial were presented at the ESMO congress 2020 [27]. In the typical/atypical LCs cohort (C1), the clinical benefit rate (CBR) at 9 months was 7.4%, with a higher expression of PD-L1 being associated with a better response. Indeed, the overall response rate (ORR) was 16% and 0% (*p* = 0.033) in patients with PD-L1-positive vs. PD-L1-negative tumours, respectively. Please read the RCTs subsection for further details about the study design.

DART (Dual Anti-CTLA-4 and Anti-PD-1 Blockade in Rare Tumours, NCT02834013) is a multicentre, open label, multiple cohort, phase II study of ipilimumab and nivolumab for rare malignancies. Although the study completion date is October 2023, patient accrual closed in December 2019 for LCs. Clinical data from the non-pancreatic neuroendocrine cohorts of the trial have been recently published [28]. Patients received ipilimumab IV over 60 min on day 1 and nivolumab IV over 30 min on days 1, 15, and 29 of a 42-day cycle in the absence of disease progression or unacceptable toxicity. Imaging studies were performed at baseline, week 8, week 16, week 24, and then every 12 weeks until disease progression, as per the RECIST 1.1 criteria. Notably, three patients with AC (P6, P12, and P24) were enrolled in the trial and all of them showed stable disease (SD; 0% ORR) with <5 months PFS [28,29].

CA209-538 (NCT02923934) is a prospective multicentre clinical trial investigating combined ipilimumab and nivolumab therapy in patients with advanced rare cancers. Preliminary data were published in February 2020 [30]. So far, one patient with TC and nine patients with AC were included in the study population. The patient with TC had SD as his best response and discontinued the treatment prior to the 24-week restaging scan due to an intervening adverse event (AE). Instead, three out of nine (33%) patients with AC achieved sustained responses, including one partial response (PR; >20 months) and two CRs (>25 and >26 months, respectively) [30]. Please read the RCTs subsection for further details about this trial.

Nestor et al. reported the case of a 40-year-old man with chemotherapy-refractory, recurrent metastatic AC showing prompt symptom relief and sustained partial radiological response with immune checkpoint blockade [31]. Notably, the tumour had been defined as PDL-1-negative, microsatellite-stable, and TMB-low based on a mediastinal lymph node biopsy. The patient completed four cycles of treatment with ipilimumab IV 3 mg/kg and nivolumab IV 1 mg/kg, after which he was started on nivolumab maintenance therapy at a flat dose of 240 mg IV every 2 weeks. ICI therapy was well tolerated, except for grade 2 pruritic dermatitis, which was successfully treated with a short course of prednisone, hydroxyzine, gabapentin, and hydrocortisone cream.

NCT03095274 (A phase II study of durvalumab (MEDI4736) plus tremelimumab for the treatment of patients with advanced neuroendocrine neoplasms of gastroenteropancreatic or lung origin (the DUNE Trial)) is a prospective, multi-centre, open-label, stratified, exploratory study evaluating the efficacy and safety of durvalumab plus tremelimumab in different cohorts of patients with advanced/metastatic, histologically confirmed, WHO 2010 G1/G2 NETs of pancreatic, gastrointestinal, and lung origins, and G3 neoplasms of GEP or unknown primary site (excluding lung primaries) being in progression after previous therapies. The dosing regimen consists of durvalumab, 1500 mg every four weeks (equivalent to 20 mg/kg every four weeks) for 12 months, in combination with tremelimumab 75 mg every four weeks (equivalent to 1 mg/kg every four weeks) for up to four doses/cycles. The trial is designed to evaluate CBR as a primary outcome, assessed by the RECIST 1.1 criteria. The ORR, DOR, PFS, and response status are secondary objectives, assessed by the immune-related RECIST criteria [32]. OS and toxicities are also evaluated as secondary endpoints. Moreover, the baseline tumour and biochemical markers are evaluated as predictors of the response of durvalumab plus tremelimumab therapy. The study started in April 2017, with an estimated enrolment of 126 participants. The actual status is “recruiting”, with an estimated date of completion of April 2022. Preliminary results were presented at the ESMO congress 2020, as detailed above.

NCT02923934 (a phase II clinical trial evaluating ipilimumab and nivolumab in combination for the treatment of rare gastrointestinal, neuro-endocrine and gynaecological cancers) is a prospective multicentre clinical trial investigating the combination of ipilimumab with nivolumab in patients with advanced rare cancers. Patients are administered ipilimumab IV at a dose of 1 mg/kg and nivolumab IV 3 mg/kg every three weeks for four doses, followed by nivolumab IV 3 mg/kg every two weeks until disease progression, the development of unacceptable toxicity, or a maximum of two years after enrolment. The response is assessed every 12 weeks based on RECIST 1.1 criteria. The primary endpoint of the study is CBR, whereas the secondary outcome is identifying a common predictive biomarker or immune signature in responder patients. The trial started in August 2017, with an estimated enrolment of 60 subjects. The current status is “active, not recruiting”, with a completion date in December 2023. The preliminary results of this trial have been published and detailed above.

NCT03420521 (an open-label, single arm phase II study of nivolumab in combination with ipilimumab in subjects with advanced neuroendocrine tumours) is a clinical trial evaluating nivolumab in combination with ipilimumab in advanced, progressive, WD non-functional NET of the pancreas, lung, or gastrointestinal tract. According to the protocol, patients receive nivolumab 240 mg IV over 60 min every 2 weeks and ipilimumab 1 mg/kg IV over 30 min every 6 weeks. One cycle includes three doses of nivolumab and one dose of ipilimumab. The primary outcome is the objective response rate, assessed by RECIST 1.1 criteria, after 6 weeks of treatment. Safety and PFS are also described as secondary measures. The trial (started in March 2018) is designed to enrol 64 patients, and the estimated study completion date is set for May 2024. The actual status is “active, not recruiting”.

### 3.5. Immune Checkpoint Inhibitors plus Chemotherapy

Sakata et al. reported the case of a 72-year-old man with advanced LC and multiple bone metastases receiving atezolizumab in combination with carboplatin plus etoposide as first-line therapy [33]. CT-guided biopsies of the primary lung tumour and a scapular metastasis resulted in the diagnosis of PD-L1-positive, microsatellite stable TC and PD-L1-negative, and microsatellite stable AC, respectively. After two cycles of chemoimmunotherapy, a CT scan of the chest showed a partial regression of the primary lung mass, while the scapular tumour was significantly enlarged. No antitumor effect was observed on other bone metastases, and therefore progressive disease (PD) was confirmed according to RECIST 1.1 criteria. Interestingly, CD8+ T cell infiltration was detected in the PD-L1-positive primary lung tumour nest, while it was limited to the stroma in the PD-L1-negative scapular metastasis, hence suggesting that the infiltrating CD8+ T cells in the tumour nest may play a key role in response to ICIs in advanced carcinoid tumours.

NCT03728361 (a phase II, multi-cohort trial of combination nivolumab and temozolomide in recurrent/refractory small-cell lung cancer and advanced neuroendocrine tumours) is a trial on the combination of nivolumab plus temozolomide in the treatment of patients with either SCLC that progressed or recurred after prior platinum-based chemotherapy and immunotherapy (cohort 1), or progressive metastatic NEN of any grade and primary site in any line of therapy (cohort 2). The treatment schedule consists of nivolumab IV on day 1 of a 28-day cycle, and temozolomide per os on days 1–5, with cycles repeated every 28 days in the absence of PD or unacceptable toxicity. The primary outcome is the objective response rate, assessed by RECIST 1.1 criteria; PFS, central nervous system PFS, OS, and safety are also evaluated as the secondary outcomes. Moreover, several exploratory objectives are analysed, including the evaluation of PD-L1 by immunohistochemistry, and the assessment of the clinical outcomes (i.e., OS) between patients with a high expression and low expression of PD-L1. The study started in December 2018 with an estimated enrolment of 53 subjects. The actual status is “recruiting, not active”, and the completion date is set for December 2022.

### 3.6. Immune Checkpoint Inhibitors plus Radiation Therapy

In 2020, Kim et al. reported the final results of a single-centre, open-label, phase I study (NCT03325816) of 177Lu-DOTA0-Tyr3-Octreotate (Lu-177) plus nivolumab in nine patients with advanced NETs of the lung, including two metastatic ACs [34]. Briefly, two different dose levels of Lu-177 (3.7 vs. 7.4 GBq every 8 weeks for four doses) were assessed in combination with nivolumab IV 240 mg every 2 weeks, and a standard 3 + 3 design was used for dose escalation. Tumour imaging was performed every 8 weeks, and the response was evaluated using the RECIST 1.1 criteria. The two patients with AC experienced no dose-limiting toxicities and had SD as the best response. One of them (patient 7) completed four cycles of Lu-177 and showed progression in the liver, where the lesions had a poor 68Gallium-DOTATATE uptake, while the other lesions were stable. The SSTR2 expression was 60% on the archival liver tissue, obtained at the time of initial diagnosis. The other patient with AC (patient 4) received three cycles of Lu-177 before progression. The SSTR2 expression was 10% on the archival lung tissue obtained at the time of the initial diagnosis. The tumour PD-L1 expression was negative in both the patients.

NCT03110978 (phase II randomized clinical trials comparing immunotherapy plus stereotactic ablative radiotherapy (I-SABR) versus SABR Alone for Stage I, selected stage IIa, or isolated lung parenchymal recurrent non-small cell lung cancer: I-SABR) is a trial evaluating the effects of stereotactic body radiation therapy with or without nivolumab in patients with early stage (I-IIA) non-small cell lung cancer or recurrent cancer, including AC. The study is based on the rationale that stereotactic body radiation therapy, delivering high biologically effective radiation doses, can kill cancer cells, release tumour-associated antigens, and activate tumour-specific T cells, thereby functioning as a cancer-specific vaccine in situ. Patients are randomized to arm I with SABR (biological effective dose > 100 Gy) over 1–2 weeks, or to arm II with SABR over 1–2 weeks plus nivolumab. Beginning within 36 h before or after the first fraction of stereotactic body radiation therapy, patients also receive nivolumab IV over 30 min on day 1. Nivolumab is repeated with cycles of 4 weeks for up to 12 weeks in the absence of PD or unacceptable toxicity. The primary endpoint is the event-free survival, with events defined as local recurrence, regional recurrence, distant metastasis, secondary malignancy (including lung cancer), and death, from the randomization date up to 5 years. OS, safety, and analyses of immunological markers are assessed as the secondary measures. The study, which started in June 2017, is estimated to enrol 140 patients, and the completion date is set for June 2022. The status is presently “recruiting”.

### 3.7. Immune Checkpoint Inhibitors plus Tyrosine Kinase Inhibitors

NCT03074513 (a phase II, single-arm open-label study of the combination of atezolizumab and bevacizumab in rare solid tumors) is a trial on the combination of atezolizumab and bevacizumab in patients with rare solid tumours, including TC and AC. Patients receive atezolizumab and bevacizumab IV over 60 min on day 1. Courses repeat every 21 days in the absence of disease progression or unacceptable toxicity. The trial is designed to evaluate the objective response rate, assessed by RECIST 1.1, as the primary endpoint. Several other efficacy and safety outcomes are evaluated as the secondary endpoints, including the objective response, assessed by immune-modified RECIST [33], DOR, PFS, OS, and AEs. The study is also aimed at identifying predictive and prognostic factors, biomarkers associated with resistance to therapy, and biomarkers associated with susceptibility to developing AEs. The study started in March 2017, and it is estimated to enrol 160 patients. Presently, the trial status is “active, not recruiting”, with an estimated date of completion of March 2021.

NCT04197310 (phase II trial of cabozantinib in combination with nivolumab for advanced carcinoid tumours) is a study aimed to evaluate the objective response rate, assessed by RECIST 1.1 criteria, after the combination of nivolumab with cabozantinib in adult patients (18 years and older) affected by unresectable or metastatic WD NET of a non-pancreatic origin (estimated enrolment 35 patients). Nivolumab is given at a dose of 240 mg every 14 days through IV, and cabozantinib is administered at the dose of 40 mg orally, once daily. The secondary outcomes are PFS, OS, ORR, and toxicity. The study started in July 2019, with the estimated study completion date being December 2022. The actual status of the study is “recruiting”.

NCT04579757 (surufatinib in combination with tislelizumab in subjects with advanced solid tumours) is an open-label, phase Ib/II study evaluating surufatinib per os in combination with tislelizumab 200 mg IV every 3 weeks in patients with locally advanced/metastatic solid tumours, including low-/intermediate-grade NETs of thoracic origin, who have progressed on or are intolerant to standard therapies. The study consists of two parts: part 1 (dose finding) is aimed to determine the recommended phase 2 dose (RP2D) and/or the maximum tolerated dose of surufatinib, whereas part 2 (dose expansion) is intended to assess the efficacy of surufatinib at RP2D in combination with tislelizumab. The incidence of the dose limiting toxicity and objective response rate are the primary outcomes in part 1 and part 2, respectively. The secondary outcomes of the study are: PFS, OS, disease control rate, DoR, CBR, time to response, AEs, and maximum plasma concentrations of surufatinib and tislelizumab with blood sampling. The study started in October 2020 with the target of enrolling 135 subjects. The actual status is “recruiting” and the completion date is set for April 2023.

## 4. Discussion

Our review shows there is some preliminary evidence of efficacy for the currently approved ICIs in the treatment of advanced and/or metastatic LCs, with the best results being reported for combined nivolumab plus ipilimumab and durvalumab plus tremelimumab regimens. This finding is in line with previously published data showing both increased response rates and median survival times with the combined anti-PD-1 and anti-CTLA-4 blockade compared to single agent anti-PD-1 treatment in patients with advanced melanoma; renal cell carcinoma, microsatellite instable colorectal cancer and subsets of non-small cell lung cancer; and increased response rates in other difficult to treat cancer types such as mesothelioma, sarcoma, and esophagogastric cancers [35,36,37]. In fact, when administered as a monotherapy, both CTLA-4 and PD-1 blockers are ineffective in a valuable fraction of patients, who may conversely respond to the simultaneous inhibition of the two pathways [38]. A synergistic mechanism due to different sites of action has been initially suggested as a reason for the greater efficacy of combined anti-CTLA-4 and anti-PD-1 regimens. Indeed, while CTLA-4 is involved in the regulation of T lymphocytes activation in lymphoid tissues and in the suppression of dendritic cells activity via Treg cells, PD-1/PD-L1 interaction inhibits the activation of T cells and natural killer cells in peripheral tissues, in this way favouring Treg cell differentiation [37]. However, preclinical studies and clinical evidence also highlight that the two ICI combination therapies elicit a unique molecular signature. For example, the production of some cytokines (CXCL10 and IL-1a) is more pronounced in patients treated with nivolumab plus ipilimumanb compared with the single agents, while other cytokines (i.e., CXCL-8) are only induced when a combined treatment is administered [39]. In particular, the chemokine CXCL-8 may induce immune infiltration in tumours and account for the response to ICIs also in the absence of pre-existing immune infiltration and PD-L1 expression on tumour cells [40]. Moreover, it has been reported that CTLA-4 inhibition favours the expansion of PD-1-expressing tumour-infiltrating lymphocytes, whereas the inhibition of PD-1 leads to the upregulation of CTLA-4 in these cells. As a consequence, the antitumor activity of anti-CTLA-4 antibodies is reduced by anti-CTLA-4-induced activation of PD-1/PD-L1 signalling in tumour-specific T effector cells, and, on the other side, the efficacy of PD-1 inhibition is limited by incomplete activation of cytotoxic T cells due to anti-PD-1 induced CTLA-4 overexpression [41]. As a result, only the combined administration of anti-CTLA-4 and anti-PD-1 antibodies may allow for complete restoration of the antitumor immune response. In the near future, the results of one more trial evaluating nivolumab plus ipilimumab combination therapy (NCT03420521) are expected to give further strength to these observations in patients with LCs (Table 2).

Importantly, treatment benefits have been observed both in PD-L1-positive and PD-L1-negative LCs. Experience with other types of cancers (e.g., melanoma) confirms that the PD-L1 expression is not a completely reliable predictive biomarker of response to ICIs, although a positive staining increases the likelihood of favourable responses. It has been reported that the results of PD-L1 staining may vary with diverse immunohistochemistry assays, and misclassification of the PD-L1 status could explain the poor ability of the response to ICI therapy for some patients [42,43]. Heterogeneous immune marker expression in primary and metastatic lesions may be another reason for variable therapeutic responses [44,45]. Of note, discordance of PD-L1 status between the primary lung tumour and metastatic bone deposits was associated with disease progression at follow-up in the above-mentioned case report by Sakata et al. [33]. When feasible, analysis of both the primary tumour mass and its secondary foci could therefore enable a better prediction of favourable outcomes with ICIs for an individual patient.

The role of combined schemes of ICIs and other antitumor treatments is emerging. Indeed, systemic chemotherapy, radiation therapy, and tyrosine kinase inhibitors may all exert a modulatory effect in the tumour microenvironment, in this way enhancing the therapeutic potential of immune checkpoint blockade [46,47,48,49,50,51]. As it was above mentioned, Lu-177 plus nivolumab showed signs of antitumor activity in a phase I study of advanced lung NENs including two metastatic ACs, with stronger uptake of 68Gallium-DOTATATE resulting in greater efficacy [34]. Furthermore, five RCTs of anti-PD-1/PD-L1 agents plus temozolomide (NCT03728361), stereotactic body radiation therapy (NCT03110978), cabozantinib (NCT04197310), bevacizumab (NCT03074513), or surufatinib (NCT04579757) are active and intended to recruit patients with LCs (Table 2), thereby revealing the great interest of researchers in this field.

New opportunities could come from the not yet approved, high-affinity, anti-PD1 humanized IgG4 antibody spartalizumab. In a phase I, multicenter, open-label study (NCT02404441) enrolling patients with locally advanced and/or metastatic solid tumours that had progressed on standard therapy, were intolerant to therapy, or for whom no standard therapy existed, one patient with PD-L1-negative metastatic AC achieved PR while on spartalizumab at a dose of 3 mg/kg every 2 weeks [52]. The DOR was 8.5 months, after which the patient developed PD. The best percentage decrease from baseline in the sum of the target lesion diameters was 77% by RECIST V.1.1, and responses were seen in multiple lesions either in the liver, in the pleura, and in lymph nodes. In line with this finding are the results of a phase II, single-arm, open-label, multicenter study (NCT02955069) that investigated the antitumor activity of spartalizumab 400 mg every 4 weeks in WD NETs of a gastrointestinal, pancreas, and thoracic origin and poorly differentiated GEP-neuroendocrine carcinomas, which had progressed with available treatments [53]. The thoracic cohort included six patients with TC and 24 patients with AC, of which five achieved PR as their best response per RECIST criteria.

Importantly, the results of both prospective trials and case studies suggest that checkpoint blockade might exert a higher antitumor activity in patients with LCs than with low-grade WD GEP-NENs [26,53,54]. However, it is important to highlight that available evidence on the efficacy of ICIs in advanced LCs mainly applies to ACs, with TCs requiring even further investigation.

## 5. Conclusions

Data about the use of ICIs in LCs are encouraging, and the number of pertinent RCTs indicate the growing interest of researchers in this field. This fervid activity will help to clarify whether these drugs, which have already shown outstanding results for other types of cancer, may represent a new arrow in the quiver in favour of patients with LC, and to define their (possible) position in the treatment algorithm.

## Figures and Tables

**Table 1:** Immune checkpoint inhibitors approved for human use.

Drug	Molecular Target	Antibody Description	US FDA Approved Indications	EU/EMA Approved Indications	China NMPA Approved Indications
Atezolizumab	PD-L1	Humanized monoclonal antibody (IgG1-kappa)	Urothelial carcinoma, NSCLC, SCLC, HCC, melanoma	Urothelial carcinoma, NSCLC, SCLC, TNBC, HCC	SCLC, HCC
Avelumab	PD-L1	Fully human monoclonal antibody (IgG1-lambda)	MCC, urothelial carcinoma, RCC	MCC, urothelial carcinoma, RCC	Not approved
Camrelizumab	PD-1	Humanized monoclonal antibody (IgG4-kappa)	Not approved	Not approved	NSCLC, HCC, esophageal cancer, cHL, NPC
Cemiplimab	PD-1	Fully human monoclonal antibody (IgG4-kappa)	CSCC, BCC, NSCLC	CSCC, BCC, NSCLC	Not approved
Durvalumab	PD-L1	Fully human monoclonal antibody (IgG1-kappa)	NSCLC, SCLC	NSCLC, SCLC	NSCLC, SCLC
Ipilimumab	CTLA-4	Fully human monoclonal antibody (IgG1-kappa)	Melanoma, RCC, CRC, HCC, NSCLC, MPM	Melanoma, RCC, NSCLC, MPM, dMMR CRC, MSI-H CRC	melanoma, RCC, MSI-H or dMMR CRC
Nivolumab	PD-1	Fully human monoclonal antibody (IgG4-kappa)	Melanoma, NSCLC, MPM, RCC, cHL, HNSCC, urothelial carcinoma, MSI-H or dMMR CRC, HCC, ESCC	Melanoma, NSCLC, MPM, RCC, cHL, HNSCC, urothelial carcinoma, MSI-H or dMMR CRC, ESCC, esophageal cancer, GEJC, gastric cancer	Gastric cancer, NSCLC, HNSCC
Pembrolizumab	PD-1	Humanized monoclonal antibody (IgG4-kappa)	melanoma, NSCLC, SCLC, HNSCC, cHL, PMBCL, urothelial carcinoma, MSI-H or dMMR cancer, MSI-H or dMMR CRC, gastric cancer, esophageal cancer, cervical cancer, HCC, MCC, RCC, endometrial carcinoma, TMB-H cancer, CSCC, TNBC	Melanoma, NSCLC, cHL, urothelial carcinoma, HNSCC, RCC, CRC, esophageal cancer, TNBC, endometrial carcinoma	Gastric cancer, esophageal cancer, GEJC, ESCC, NSCLC, melanoma
Penpulimab	PD-1	Humanized monoclonal antibody (IgG1-kappa)	Not approved	Not approved	cHL
Sintilimab	PD-1	Fully human monoclonal antibody (IgG4-kappa)	Not approved	Not approved	Gastric cancer, GEJC, cHL, NSCLC, SCLC, HCC
Tislelizumab	PD-1	Humanized monoclonal antibody (IgG4-kappa)	Not approved	Not approved	NSCLC, HCC, cHL, urothelial carcinoma
Toripalumab	PD-1	Humanized monoclonal antibody (IgG4-kappa)	Not approved	Not approved	NPC, melanoma
Zimberelimab	PD-1	Fully Human monoclonal antibody (IgG1-kappa)	Not approved	Not approved	cHL

NSCLC, non-small cell lung cancer; SCLC, small cell lung cancer; HCC, hepatocellular carcinoma; TNBC, triple-negative breast cancer; MCC, Merkel cell carcinoma; RCC, renal cell carcinoma; cHL, classical Hodgkin lymphoma; NPC, nasopharyngeal carcinoma; CSCC, cutaneous squamous cell carcinoma; BCC, basal cell carcinoma; CRC, colorectal cancer; MPM, malignant pleural mesothelioma; MSI-H, microsatellite instability-high; dMMR, mismatch repair deficient; HNSCC, head and neck squamous cell cancer; ESCC, esophageal squamous cell carcinoma; GEJC, gastroesophageal junction cancer; PMBCL, primary mediastinal large B-cell lymphoma.

**Table 2:** Ongoing registered clinical trials of immune checkpoint inhibitors in advanced cancers that are expected to recruit patients with lung carcinoids.

ClinicalTrials.gov Identifier	First Posted	Molecule	Trial Name	Phase	Assigned Intervention	Primary Outcome	Estimated Study Completion Date	Trial Status
NCT02628067	11 December 2015	Pembrolizumab	A clinical trial of pembrolizumab (MK-3475) evaluating predictive biomarkers in subjects with advanced solid tumors (KEYNOTE 158)	Phase II	Arm I: pembrolizumab 200 mg IV on day 1 of each 3-week cycle for up to 35 administrations (up to approximately 2 years of treatment); arm II: pembrolizumab 400 mg every 6 weeks for up to 18 administrations (up to approximately 2 years of treatment)	Objective response rate (time frame: up to approximately 2 years)	18 June 2026	Recruiting
NCT02923934	5 October 2016	Nivolumab + ipilimumab	A phase II clinical trial evaluating ipilimumab and nivolumab in combination for the treatment of rare gastrointestinal, neuro-endocrine and gynaecological cancers	Phase II	Nivolumab at 3 mg/kg + ipilimumab at 1 mg/kg concurrently every 3 weeks for 4 doses followed by nivolumab only at 3 mg/kg every 2 weeks until progression (up to 48 total doses of nivolumab)	CBR (time frame: at 12 weeks following randomization then every 6 weeks until disease progression)	December 2023	Active, not recruiting
NCT03074513	8 March 2017	Atezolizumab + bevacizumab	A phase II, single-arm open-label study of the combination of atezolizumab and bevacizumab in rare solid tumors	Phase II	Atezolizumab + bevacizumab IV over 60 min on day 1. Courses repeat every 21 days in the absence of disease progression or unacceptable toxicity	Objective response (time frame: up to 4 years)	31 March 2021	Active, not recruiting
NCT03095274	29 March 2017	Durvalumab + tremelimumab	A phase II study of durvalumab (MEDI4736) plus tremelimumab for the treatment of patients with advanced neuroendocrine neoplasms of gastroenteropancreatic or lung origin (the DUNE Trial)	Phase II	Durvalumab, 1500 mg every 4 weeks for 12 months + tremelimumab 75 mg every 4 weeks for up to 4 doses/cycles	CBR (time frame: 9 months)	April 2022	Recruiting
NCT03110978	12 April 2017	Nivolumab + SABR	Phase II randomized clinical trials comparing immunotherapy plus stereotactic ablative radiotherapy (I-SABR) versus SABR alone for stage I, selected stage IIa, or isolated lung parenchymal recurrent Non-small cell lung cancer: I-SABR	Phase II	Arm I: SABR over 1–2 weeks; arm II: SABR over 1–2 weeks + nivolumab IV over 30 min on day 1. Cycles with nivolumab repeat every 4 weeks for up to 12 weeks	Event-free survival (time frame: from the randomization date, assessed up to 5 years)	30 June 2022	Recruiting
NCT03420521	5 February 2018	Nivolumab + ipilimumab	An open-label, single arm phase II study of nivolumab in combination with ipilimumab in subjects with advanced neuroendocrine tumors	Phase II	Nivolumab 240 mg IV over 60 min every 2 weeks + ipilimumab 1 mg/kg IV over 30 min every 6 weeks	Objective response rate [time frame: 6-weeks post-intervention]	1 May 2024	Active, not recruiting
NCT03728361	2 November 2018	Nivolumab + temozolomide	A phase II, multi-cohort trial of combination nivolumab and temozolomide in recurrent/refractory small-cell lung cancer and advanced neuroendocrine tumors	Phase II	Nivolumab IV on day 1 of a 28-day cycle + temozolomide PO on days 1–5. Courses repeat every 28 days	Objective response rate [time frame: up to 3 years]	31 December 2021	Active, not recruiting
NCT04197310	13 December 2019	Nivolumab + cabozantinib	phase II trial of cabozantinib in combination with nivolumab for advanced carcinoid tumors	Phase II	Nivolumab 240 mg, IV, day 1 and 15 of a 28-day cycle + cabozantinib 40 mg, orally, daily for a 28 day cycle	Objective response rate (time frame: 2 years)	26 December 2022	Recruiting
NCT04579757	8 October 2020	Tislelizumab + surufatinib	Surufatinib in combination with tislelizumab in subjects with advanced solid tumors	Phase Ib/II	Part 1 (dose escalation): surufatinib PO once daily + tislelizumab 200 mg IV every 3 weeks; Part 2 (dose expansion): surufatinib at the recommended phase 2 dose as determined in Part 1 + 200 mg tislelizumab IV, every 3 weeks	Part 1: Incidence of dose limiting toxicity (time frame: up to 60 days) Part 2: objective response rate (time frame: up to 2 years)	27 February 2023	Recruiting

CBR, clinical benefit rate; IV, intravenously; PO, per os; SABR, stereotactic ablative radiotherapy.

## Data Availability

No new data were created or analysed in this study. Data sharing is not applicable to this article.
